# A tricky case of cardiogenic shock: Diagnostic challenges in the COVID‐19 era

**DOI:** 10.1002/ccr3.3546

**Published:** 2020-11-20

**Authors:** Vasileios Sachpekidis, Chris Adamopoulos, Antonios Datsios, Lampros Mosialos, Nikolaos Stamatiadis, Christos Gogos, Vasileios Poulianitis, Othonas Galanos, Sofia Stratilati, Ioannis Styliadis, Petros Nihoyannopoulos

**Affiliations:** ^1^ Department of Cardiology Papageorgiou Hospital Thessaloniki Greece; ^2^ Department of Cardiology Ippokrateion Hospital Thessaloniki Greece; ^3^ Department of Cardiothoracic Surgery Papageorgiou Hospital Thessaloniki Greece; ^4^ Department of Radiology Papageorgiou Hospital Thessaloniki Greece; ^5^ Department of Cardiovascular Sciences Hammersmith Hospital Imperial College London London UK

**Keywords:** cardiogenic shock, COVID‐19, handheld echocardiography, intrapericardial hematoma, myocardial infarction, myocardial wall rupture

## Abstract

Myocardial wall rupture should be considered in patients presenting with hypotension and STEMI especially of delayed onset. Diagnosing this entity in the COVID‐19 era can be challenging—handheld echocardiography may aid toward this end.

## Graphical abstract

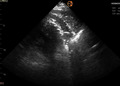

## 1 INTRODUCTION

We describe the case of a 70‐year‐old obese patient that presented in shock, 48 hours after suffering inferior ST‐elevation myocardial infarction (STEMI). The patient avoided coming earlier to the hospital due to fear of Coronavirus Disease 2019 (COVID‐19) infection. Clinical assessment and bedside echocardiography suggested contained myocardial wall rupture that was confirmed subsequently with computed tomography (CT). Urgent cardiac surgery was performed resulting in good outcome.

We discuss diagnostic and treatment challenges of this rare but life‐threatening complication of myocardial infarction, which are augmented when coping with such cases in the COVID‐19 era.

## 2 CASE DESCRIPTION

A 70‐year‐old obese (Body Mass Index – BMI ~35 kg/m^2^) male presented to the ER due to syncope while seated. He reported chest pain radiating to the left arm during the last 2 days which has subsided a few hours before presentation. Noticeably, the patient avoided coming earlier to the hospital due to fear of COVID‐19 infection. The patient was ex‐smoker (he quit at the age of 40) with no other medical history.

On admission the patient was in shock with cold extremities, blood pressure of 81/60 mm Hg and heart rate of 80 beats/min. He was lying flat and had dilated jugular veins. There was no audible cardiac murmur, and his lung fields were clear. He was afebrile.

His electrocardiogram (ECG) showed sinus rhythm and ST elevation with T‐wave inversion in the inferior leads, suggestive of recent inferior myocardial infarction. Troponin was elevated confirming the diagnosis of myocardial infarction.

### 2.1 Differential diagnosis

Given the patient's clinical presentation (shock with dilated jugular veins, no cardiac murmur, and clear lung fields), ECG findings (ST elevation in the inferior leads) and troponin elevation, cardiogenic shock due to right ventricular (RV) myocardial infarction was our most likely diagnosis. Ventricular septal rupture and papillary muscle rupture were unlikely due to the absence of cardiac murmur, dyspnea, and pulmonary crackles. Other types of shock (hypovolemic or distributive) were also excluded based on clinical presentation and dilation of jugular veins.

### 2.2 Investigations and treatment

**FIGURE 1:**
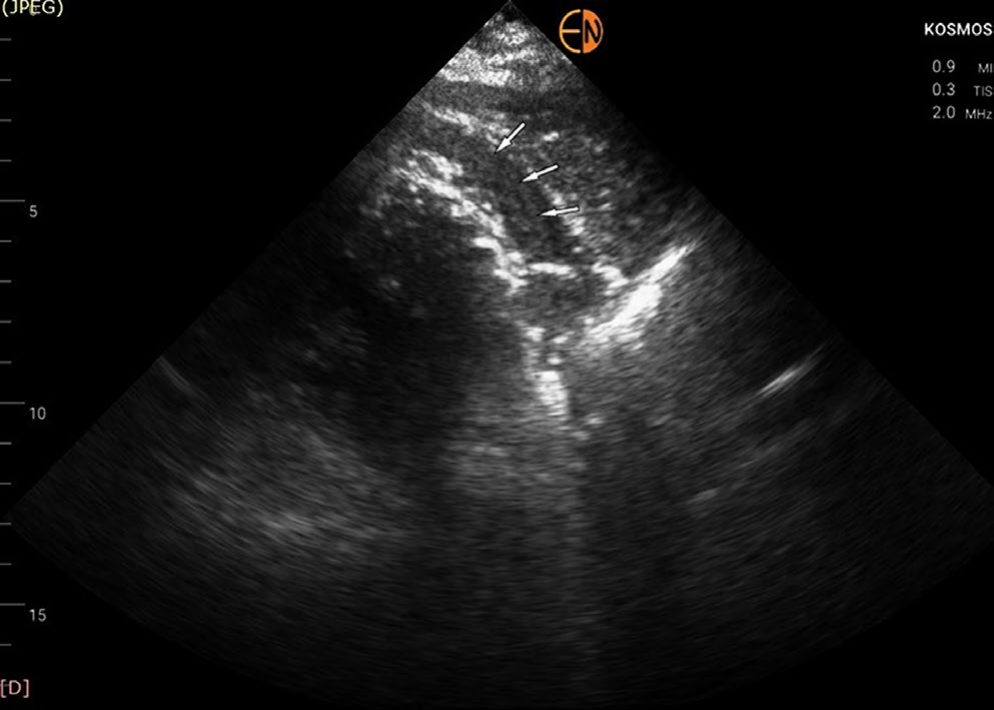
Modified parasternal short‐axis view showing the presence of intrapericardial hematoma (white arrows)

**FIGURE 2:**
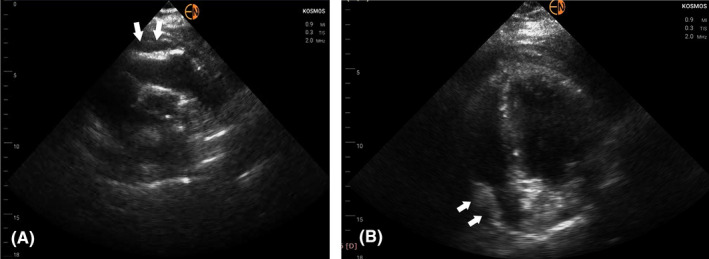
A, Parasternal short‐axis view at the level of aortic valve demonstrating compression of the RV (white arrows); B, Apical four‐chamber view showing compression of the right atrium (white arrows)

Due to unavailability of the standard cart‐based echo machine in the Coronary Care Unit (it was used for scanning a confirmed COVID‐19 patient), a transthoracic echocardiogram with the use of a handheld device was immediately performed (Kosmos, Echonous Inc). To our surprise the RV had normal systolic function, a finding strongly opposing our main clinical hypothesis. The left ventricle (LV) had only mildly impaired systolic function (Ejection Fraction ~40%‐45%) due to inferior and inferolateral wall akinesia, confirming the diagnosis of inferolateral myocardial infarction. No valvular abnormality or ventricular septal rupture was seen. There appeared to be an intrapericardial hematoma (modified short‐axis view in Figure 1—white arrows; Video S1) which, combined with the presence of compression of the right heart chambers (Figure 2A,B—white arrows) and the dilated inferior vena cava led us to the presumptive diagnosis of cardiac tamponade due to contained myocardial rupture resulting from the recent myocardial infarction. All these findings were confirmed when we scanned the patient with the standard cart‐based echo machine (Epic, Philips Inc) as soon as it became available (the added to scanning time for sterilizing the machine is not negligible)—Video S2 and Video S3. Pulsus paradoxus was evident when a left radial arterial line was placed further supporting our diagnosis.

Patient was temporarily hemodynamically stabilized with vasopressors (noradrenaline) and fluids and cardiothoracic surgical consultation was promptly asked. Antiplatelet (except from aspirin which had already been given in the ER) and heparin treatment was withheld.

**FIGURE 3:**
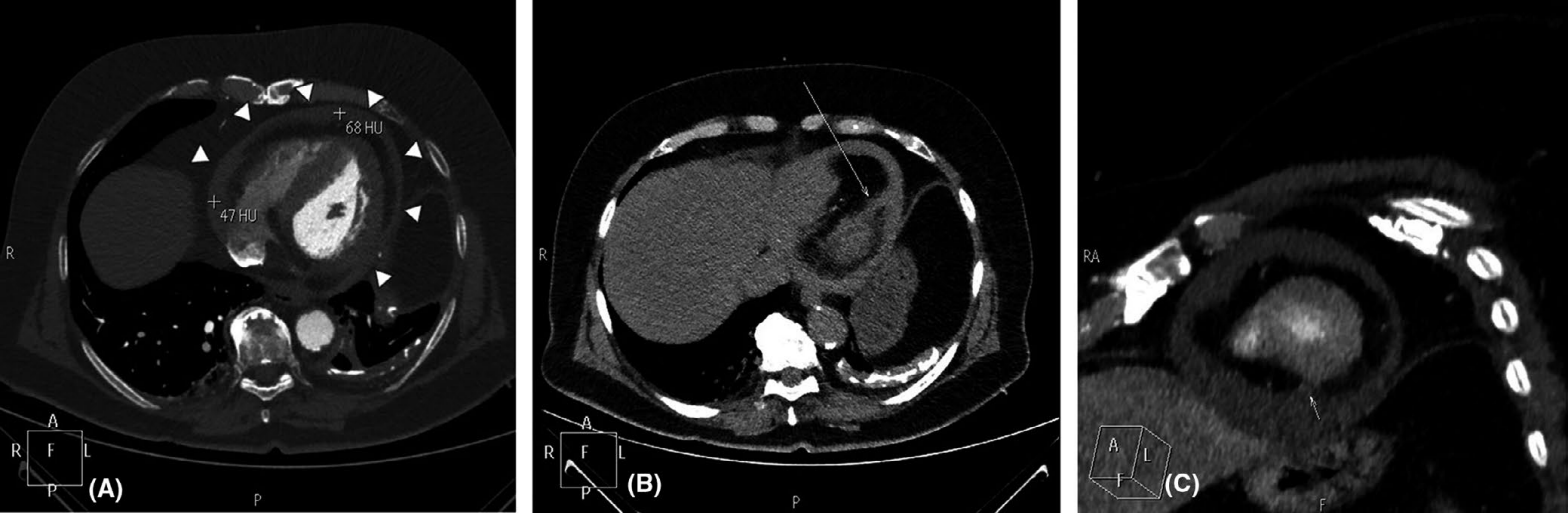
A, Axial CT imaging showing hemopericardium (white arrowheads); (B and C) Reconstructed CT images showing the site of perforation in the inferolateral wall (thin white arrows)

**FIGURE 4:**
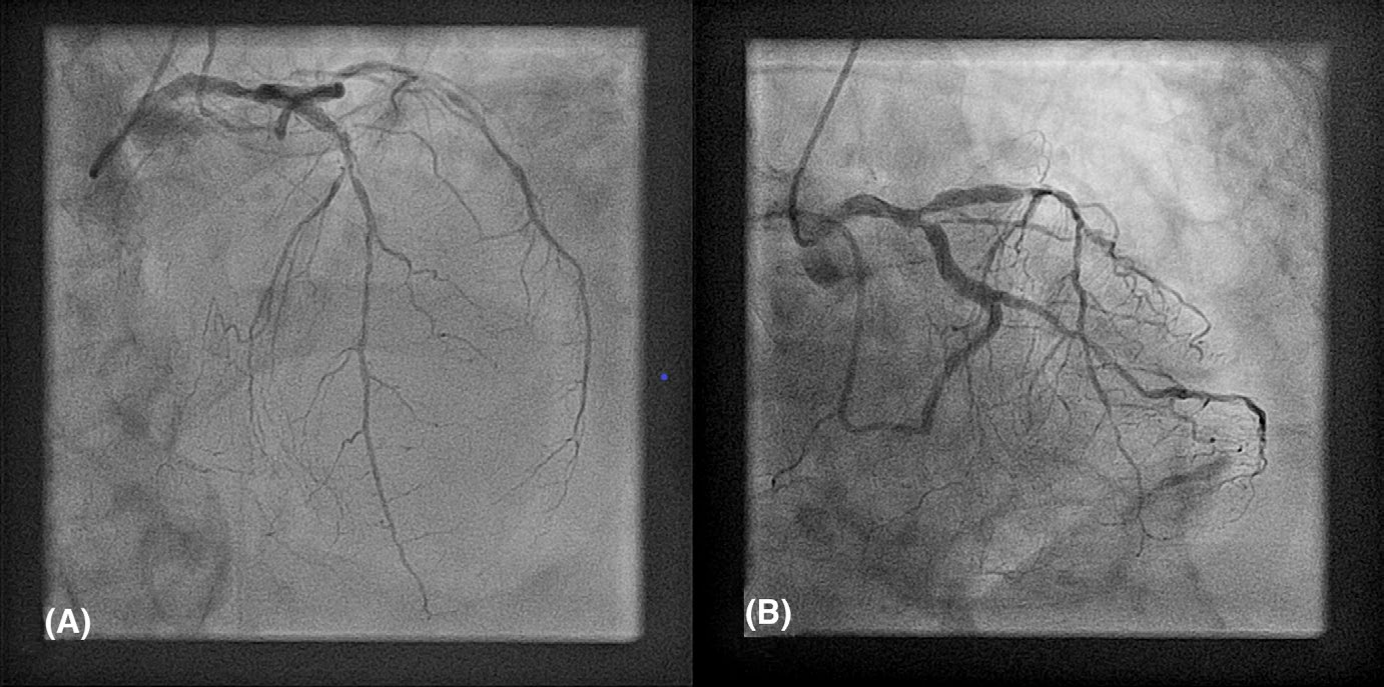
A, Right anterior oblique cranial view showing severe left anterior descending artery stenosis; B, Right anterior oblique caudal view showing occluded left circumflex artery

A CT aortography performed immediately after, excluded the presence of acute aortic syndrome (which can present in a similar way) and confirmed the presence of hemopericardium based on the Hounsfield Unit values of the pericardial effusion (Figure 3A—white arrowheads). Postprocessing of the CT images was able to reveal the site of rupture in the inferolateral wall (Figure 3B,C—thin white arrows). Cardiac catheterization performed from the right radial artery showed an occluded dominant left circumflex (LCx) artery at its mid portion and severe left anterior descending (LAD) artery stenosis (Figure 4A,B). The right coronary artery (RCA) was a small, hypoplastic vessel.

**FIGURE 5:**
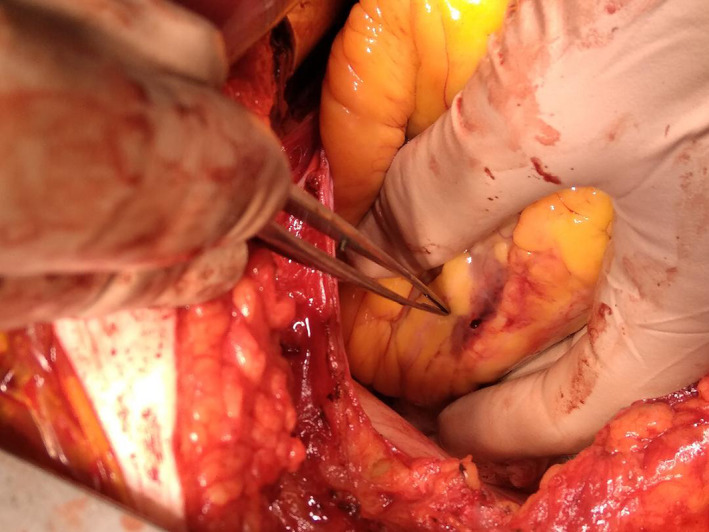
Intraoperative photograph showing the perforation of the inferolateral wall

Based on these findings the patient was immediately taken to the operating table; the presence of both intrapericardial hematoma and myocardial wall rupture of the inferolateral wall was confirmed (Figure 5). Rupture was closed with a pericardial patch and a left internal mammary artery (LIMA) graft to the LAD was placed. The right coronary artery was a small, hypoplastic vessel and the distal LCx was a relatively small caliber vessel and not considered a suitable target for grafting.

### 2.3 Outcome and follow‐up

The patient made a quick and uneventful recovery and was discharged home on day 8 feeling well. On his discharge echocardiogram, the LV systolic function was only mildly reduced due to inferior and inferolateral wall akinesia. No pericardial effusion was present.

## 3 DISCUSSION

Myocardial wall rupture after myocardial infarction is rare and contemporary data from a large US database involving more than 9 million STEMI and NSTEMI hospitalizations reported an incidence of 0.01%.^1^, ^2^ Prognosis of these patients is dismal with 39% in hospital mortality in the SHOCK trial.^3^ The early use of reperfusion strategies and adjunct medical therapies appear to decrease cardiac rupture but in our case the delay in seeking medical help due to fear of COVID‐19 infection deprived him of the benefit of timely coronary reperfusion. Recent data from Italy confirm the reduced admissions for STEMI during the COVID‐19 outbreak with a parallel more than three times increase in complications and fatality rate.^4^

Early rupture (<72 hours) as in our patient is usually characterized by an abrupt slit like tear in the infarction area, in contradiction to infarct expansion which is the main feature of late (>4 days) ruptures.^5^ Establishing risk factors for LV free wall rupture is very difficult due to the small number of affected patients. In the contemporary percutaneous intervention era risk factors related to mechanical complications in STEMI patients are older age, female sex, white race, chronic kidney disease, and the presence of valvular heart disease.^1^

Clinical presentation of myocardial wall rupture varies. More than half of cases occur within the first 5 days after myocardial infarction just like our patient.^5^, ^6^ Complete myocardial rupture leads very quickly to hemopericardium and death due to cardiac tamponade. The sudden appearance of pulseless electrical activity in a patient with acute myocardial infarction in the absence of heart failure symptoms should strongly raise the suspicion of complete myocardial wall rupture.^7^ In case of incomplete/subacute rupture intrapericardial thrombus and the pericardium seals the perforation offering a variable time window for diagnostic and treatment interventions. However, repetitive bleeding to the pericardial sac can occur, causing progressive or recurrent tamponade. Patients with incomplete/subacute rupture usually present with recurrent chest pain, agitation, hypotension, syncope, or shock.^8^, ^9^ Our patient had all these features apart from recurrence of chest pain.

The diagnosis of suspected myocardial wall rupture is usually made with echocardiography, with most common features being the presence of pericardial effusion with or without intrapericardial thrombus/hematoma and right heart chamber compression due to tamponade.^8^, ^9^ The site of rupture is very difficult to visualize in the vast majority of patients. In our case, there was a large pericardial hematoma compressing the right heart chambers with free pericardial fluid hardly seen. Imaging was very difficult due to patient's large body habitus (ΒΜΙ ~35 kg/m^2^). To make things worse, the cart‐based standard echo machine used in the Coronary Care Unit was unavailable because it was used for scanning a confirmed, critically ill COVID‐19 patient in a nearby isolated area. We used a handheld device instead and still were able to make the correct diagnosis, in spite of the large BMI of the patient, highlighting the importance of availability of such devices for clinical decision‐making especially in emergency cases.

In our case, diagnosis was confirmed with CT imaging. If the patient is relatively stable, CT can assist in the diagnosis of myocardial wall rupture by showing extravasation of contrast in the pericardium and/or the presence of hemopericardium. It is also possible to localize the site of myocardial wall rupture and exclude ascending aorta dissection which can also cause hemopericardium. Cardiac Magnetic Resonance is usually not an option because these patients are critically ill, but it can be helpful in cases of impending cardiac rupture.^10^

Prompt recognition of myocardial rupture is important for initiation of the appropriate treatment. In patients with shock, as in our case, temporary clinical stabilization can be achieved with fluids and inotropes or vasopressors.^10^ Emergency pericardiocentesis can be lifesaving in the critically ill patient that cannot be stabilized with medical treatment.^7^, ^8^, ^9^, ^11^ If a pericardial thrombus is seen compressing the cardiac chambers, like in our case, pericardiocentecis is unlikely to be successful; this is the reason we avoided performing it. In addition, the success of the procedure can be limited by rapid clotting of blood into the set for pericardial drainage.^9^

If the patient stabilizes after pericardiocentesis and bleeding stops, a conservative approach might be justified in selected patients. However, immediate cardiac surgery should be considered in most cases. Surgical management includes placement of a pericardial patch with biological glue or epicardial sutures, providing stability. Infarctectomy with patch placement and ventricular wall reconstruction is also an option in selected cases.^10^ In our patient, a pericardial patch was placed on the site of the perforation with very good short‐term results.

### 3.1 Follow‐up

Patient was seen 2 months after discharge and is doing well. He is on dual‐antiplatelet therapy and statin. He exercises regularly with no angina or dyspnea. He is due to have a functional stress test to assess for signs of ischemia in the lateral wall and based on the findings further treatment decisions will be made.

## 4 CONCLUSIONS

Contained myocardial wall rupture should be considered in every patient with acute myocardial infarction that presents in shock, especially in the COVID‐19 era where many STEMI patients present late to the hospital due to fear of infection. Echocardiography and CT can establish the diagnosis, which if missed can lead to devastating outcomes. Urgent surgery is the treatment of choice in the majority of patients.

## CONFLICT OF INTEREST

None declared.

## AUTHOR CONTRIBUTIONS

VS: contributed to collecting clinical data, literature review, and writing of the manuscript. CA: contributed to literature review and writing of the manuscript. AD: contributed to collecting clinical and angiographic data and revision of the manuscript. LM: contributed to collecting angiographic data and revision of the manuscript. NS: contributed to collecting clinical data and revision of the manuscript. CG: contributed to collecting clinical data and revision of the manuscript. VP: contributed to collecting intraoperative data and revision of the manuscript. OG: contributed to collecting intraoperative data and revision of the manuscript. SS: contributed to collecting radiology data and revision of the manuscript. IS: contributed to literature review and revision of the manuscript. PN: contributed to literature review and revision of the manuscript.

## INFORMED CONSENT

Informed consent was obtained from the patient for publication of this case report and any accompanying images.

## Supporting information

Video S1

Video S2

Video S3

## Data Availability

The authors confirm that the data supporting the findings of this case report are available within the article and its supplementary materials.
